# Dr-FtsA, an Actin Homologue in *Deinococcus radiodurans* Differentially Affects Dr-FtsZ and Ec-FtsZ Functions *In Vitro*

**DOI:** 10.1371/journal.pone.0115918

**Published:** 2014-12-31

**Authors:** Kruti Modi, Hari S. Misra

**Affiliations:** Molecular Biology Division, Bhabha Atomic Research Centre, Mumbai, 400 085, India; University of Groningen, Groningen Institute for Biomolecular Sciences and Biotechnology, Netherlands

## Abstract

The *Deinococcus radiodurans* genome encodes homologues of divisome proteins including FtsZ and FtsA. FtsZ of this bacterium (Dr-FtsZ) has been recently characterized. In this paper, we study FtsA of *D. radiodurans* (Dr-FtsA) and its involvement in regulation of FtsZ function. Recombinant Dr-FtsA showed neither ATPase nor GTPase activity and its polymerization was ATP dependent. Interestingly, we observed that Dr-FtsA, when compared with *E. coli* FtsA (Ec-FtsA), has lower affinity for both Dr-FtsZ and Ec-FtsZ. Also, Dr-FtsA showed differential effects on GTPase activity and sedimentation characteristics of Dr-FtsZ and Ec-FtsZ. For instance, Dr-FtsA stimulated GTPase activity of Dr-FtsZ while GTPase activity of Ec-FtsZ was reduced in the presence of Dr-FtsA. Stimulation of GTPase activity of Dr-FtsZ by Dr-FtsA resulted in depolymerization of Dr-FtsZ. Dr-FtsA effects on GTPase activity and polymerization/depolymerisation characteristics of Dr-FtsZ did not change significantly in the presence of ATP. Recombinant *E. coli* expressing Dr-FtsA showed cell division inhibition in spite of *in trans* expression of Dr-FtsZ in these cells. These results suggested that Dr-FtsA, although it lacks ATPase activity, is still functional and differentially affects Dr-FtsZ and Ec-FtsZ function *in vitro*.

## Introduction

Bacterial cell division is controlled by the coordinated action of an array of proteins that constitute the divisome. Along with FtsZ, FtsA, an actin homologue in bacteria, is also an essential cell division protein. FtsA recruits FtsZ polymers to the membrane [1]. FtsA and FtsZ first co-assemble into polymers after which the negative influence of FtsA on FtsZ filament stability leads to dynamicity during cytokinesis [2]. Mechanistically, FtsA binds to the membrane through its C-terminal membrane anchoring domain that binds lipids and thus helps in tethering of Z-ring to the membrane [1], [3]. Mutations in the ATP binding region of FtsA abolish its self interaction as well as interaction with FtsZ [4]. Functional homologues of FtsA have been identified in some bacteria. For example, ZipA in *E. coli* could perform overlapping functions of FtsA [5] and a FtsA gain of function mutant could complement the loss of ZipA in *E. coli* [6]. FtsA from *E. coli* and *Thermotoga maritima* have been shown to possess two subdomains namely 1C and 2B [7], [8]. Deletion analyses have shown that the S12–S13 strands of subdomain 2B are essential for the interaction and recruitment of FtsZ to the membrane. Deletion of subdomain 2B abolishes the interaction of FtsA with FtsZ, leading to filamentation [8]. On the other hand, subdomain 1C is required for FtsA self interaction and for recruiting FtsQ and FtsN to the division ring [8]. Mostly FtsA has been shown to bind ATP and the requirement of ATP for FtsA polymerisation has been demonstrated in case of *Bacillus subtilis, Thermotoga. maritima* and *Streptococcus pneumoniae* [9]–[11]. However, ATP hydrolysis has been reported only for FtsA from *B. subtilis* [9]. The other factor that contributes to the *in vivo* functions of FtsZ and FtsA is their intracellular ratio, which is estimated to be approximately 5∶1 in both *E. coli* and *B. subtilis* [9], [12]. Any perturbation to this ratio by either over expression or deletion of any of these proteins affects cell division and causes cell elongation. However, the molecular basis of this effect is not well understood.

*Deinococcus radiodurans* is known for its extreme tolerance to various DNA damaging agents [13]–[15]. It contains an efficient DNA strand break repair machinery and protects its proteome from oxidative damage [16], [17]. *D. radiodurans* genome encodes nearly all the divisome components including early divisome proteins like FtsZ, FtsA and FtsK as well as late divisome proteins like FtsQ, FtsE and FtsW [18]. Transcriptome analysis shows that the levels of transcripts for the majority of the divisome components do not change during post irradiation recovery (PIR) [19]. Immunoblotting using antibodies against FtsZ from *D. radiodurans* (Dr-FtsZ) also shows that the levels of Dr-FtsZ do not change during PIR [20]. Dr-FtsZ is shown to be a slow GTPase that shows polymerization/depolymerization dynamics *in vitro* and localizes to division sites in *D. radiodurans* cells [20]. We observed that *E. coli* cells overexpressing Dr-FtsA or Dr-FtsZ get elongated, which could have been attributed due to cell division inhibition. Therefore, functional similarity of Dr-FtsA with FtsA from *E. coli* (Ec-FtsA) and molecular basis to cell division inhibition would be worth investigating. Here we report that Dr-FtsA interacts differently with Dr-FtsZ and Ec-FtsZ both *in vivo* and *in vitro* and also differentially regulates GTPase activity and polymerization dynamics of these enzymes *in vitro*. For instance, Dr-FtsA stimulated GTPase activity of Dr-FtsZ while it inhibited Ec-FtsZ GTPase activity, most likely by interfering with its polymerization. Although, the recombinant Dr-FtsA interacts with ATP and produces long polymeric filaments in the presence of ATP, it did not show ATPase activity. These results suggested that FtsA of *D. radiodurans* is functionally active and its interaction with Dr-FtsZ and Ec-FtsZ could affect these enzymes activity *in vivo* and *in vitro*.

## Materials and Methods

### Bacterial strains, plasmids and materials

*Deinococcus radiodurans* R1 (ATCC13939) was a gift from Professor J. Ortner, Germany [21]. The *E. coli* strains DH5α and NOVABLUE were used for cloning and *E. coli* BL21 (DE3) pLysS and TOP10 were used for the expression of recombinant proteins. *E. coli* BTH101 was used for co-expression of the respective interacting partners for *in vivo* protein-protein interaction studies. *E. coli* was grown in LB and *D. radiodurans* was grown in TGY medium with shaking at 180 rpm at 37°C and 32°C, respectively. *E. coli* vectors, pET28a(+), pBAD/HisA, p11559 [22] and pVHS559 [23] were maintained in *E. coli* DH5α. Ampicillin (100 µg/ml), kanamycin (50 µg/ml) and spectinomycin (40 µg/ml) and (75 µg/ml) for *D. radiodurans* and *E. coli*, respectively were used as required. Recombinant *E. coli* harbouring expression vectors and their derivatives were grown in the presence of antibiotics as required. All recombinant techniques were performed as previously described [24]. Antibodies against FtsZ of *D. radiodurans* were commercially produced in rabbit (MERCK Millipore, India), antibodies against FtsZ of *E. coli* were generous gift from Professor P. Ajitkumar, IISc Bangalore, and GFP and anti-his antibodies were procured from Roche Biochemical Mannheim, Germany. Molecular biology grade chemicals and enzymes were procured from Sigma Chemicals Company, USA, Roche Biochemicals, Mannheim, Germany, New England Biolabs, USA and Bangalore Genie, India. Radio-labelled nucleotides were obtained from Board of Radiation and Isotope Technology, Department of Atomic Energy, India (BRIT, India). Details of the primers used in this study are given in Table 1.

**Table 1:** List of the primers used in this study.

Sl No	Name of primer	Primer sequence	Purpose/plasmid
1	DFtsAF	5′CCGCTCGAGATGAGAGAAAACAGCATCA 3′	pBADDFtsA
2	DFtsAR	5′CCGGAATTCTCAGAACCAGTCGCGGAACA 3′	pBADDFtsA
3	EFtsAF	5′CCGCTCGAGATGATCAAGGCGACGGACA 3′	pBADEFtsA
4	EFtsAR	5′CCGGAATTCTTAAAACTCTTTTCGCAGCCA 3′	pBADEFtsA
5	DFZ25NF	5′GCGGATCCGGCAGCTATGCAAGCAGCCAGAATTC3′	pKNDFZ
6	DFZ25R	5′CGGGGTACCGCGGCCGCCTGTCCGCCGACGTTGC 3′	pKNDFZ
7	EFZNF	5′CTAGTCTAGAGGCAGCTATGTTTGAACCAATGGA 3′	pKNEFZ
8	EFZNR	5′CGCGGATCCGCGGCCGCATCAGCTTGCTTACGCA 3′	pKNEFZ
9	DFZ18F	5′GCGGATCCGGCAGCTATGCAAGCAGCCAGAATTC 3′	pUTDFZ
10	DFZ18R	5′CGGGGTACCGCGGCCGCCTGTCCGCCGACGTTGC 3′	pUTDFZ
11	DFA18CF	5′ CGCGGATCCGGCAGCTATGAGAGAAAACAGCA 3′	pUTCDFA
12	DFA18CR	5′CGGGGTACCGCGGCCGCTCAGAACCAGTCGCGGA 3′	pUTCDFA
13	EFA18CF	5′ CTAGTCTAGAGGCAGCTATGATCAAGGCGACGGA 3′	pUTCEFA
14	EFA18CR	5′CGCGGATCCGCGGCCGCTTAAAACTCTTTTCGCAG 3′	pUTCEFA
			

### Construction of expression plasmids

For purification of recombinant proteins, Dr-FtsZ expressing plasmid was constructed as described earlier [20]. Ec-FtsZ expressing plasmid is as described in [25]. Dr-FtsA and Ec-FtsA expressing plasmids were constructed in the present study. Genomic DNA of *E. coli* MG1655 and *D. radiodurans* R1 were isolated as described in [26]. *fts*A gene was PCR amplified from *E. coli* and *D. radiodurans* genomic DNA using forward primers EFtsAF and DFtsAF and reverse primers EFtsAR and DFtsAR, respectively (Table 1). PCR products were cloned at *Xho*I and *Eco*RI sites in vector pBAD/HisA under *araBAD* promoter to yield the pBADEftsA and pBADDftsA plasmids. For *in trans* expression of FtsZ in FtsA expressing cells, Dr-FtsZ was expressed as FtsZ-GFP on pFTSZGFP [20]. PCR amplified coding sequence of *gfp* was also cloned in pVHS559 at *ScaI* and *SalI* sites to give pVHSgfp. The expression of FtsZ-GFP fusion was confirmed by immunoblotting using antibodies against Dr-FtsZ. These plasmids were maintained in expression host *E. coli* MG1655. For studying protein-protein interaction *in vivo*, *fts*Z and *fts*A of *D. radiodurans* and *E. coli* were PCR amplified using gene specific primers with required restriction sites and incorporated in BTH vectors (pUT18. pUT18C and pKNT25) as given in Table 1. Details of bacterial strains and plasmids used in this study are given in Table 2.

**Table 2:** Bacterial strains and plasmids used in this study.

Materials	Description	Source
**Bacterial strains**		
*Deinococcus radiodurans* R1	Wild type strain ATCC13939	Laboratory collection
*E. coli* MG1655	Wild type	Laboratory collection
*E. coli* MG1655*fts*Z84	MG1655 ftsZ84 (Ts)*leu*::Tn10	[46]
*E. coli* BL21 (DE3)	*fhu*A2 (*lon*)*omp*T *gal*(λDE3)(*dcm*) Δ*hsd*S	Laboratory collection
*E. coli* Novablue	*end*A1*hsd*R1(rK12^−^mK12^+^)*sup*E*44thi-*1 *rec*A1 *gyr*A96 *rel*A1 *lac*F*′* [*pro*A^+^B^+^ *lac*I^q^ *ZΔM15*::*Tn*10(Tc^R^)]	NEB Inc
*E. coli* TOP10	F-*mcr*AΔ(*mrr*-*hsd*RMS-*mcr*BC) Φ80*lac*ZΔM15 Δ*lac*X74 *rec*A1 *ara*D139 Δ(*ara*A-leu)7697 *gal*U *gal*K *rps*L *end*A1 *nup*G	Invitrogen
*E. coli* BTH101	F′, *cya*-99, *ara*D139, *gal*E15, *gal*K16, *rps*L1 (StrR), *hsd*R2, *mcr*A1, *mcr*B1, *rel*A1	[47]
	**Plasmids**	
pBAD/HisA	4.1 kb, Amp^r^	Invitrogen
pBADDFtsA	∼1.5 kb *Dr-ftsA* cloned in pBAD/HisA at *Xho*I and *Eco*RI sites, 5.6 kb, Amp^r^	This study
pBADEFtsA	∼1.1 kb *Ec-ftsA* cloned in pBAD/HisA at *Xho*I and *Eco*RI sites, 5.3 kb, Amp^r^	This study
pKNT25	Origin of replication from pSU40. N-terminal fusion with T25 fragment of adenylate cyclase, 3.4 kb, Kan^r^	[47]
pUT18	*colE1* origin of replication from pUC19. N-terminal fusion with T18 fragment of adenylate cyclise. 3 kb, Amp^r^	[47]
pUT18C	*colE1* origin of replication from pUC19. C-terminal fusion with T18 fragment of adenylate cyclase. 3 kb, Amp^r^	[47]
pKNDFZ	∼1.1 kb *Dr-ftsZ* cloned in pKNT25 at *Bam*HI and *Kpn*I sites, 4.52 kb, Kan^r^	This study
pKNEFZ	∼1.1 kb *Ec-ftsZ* cloned in pKNT25 at *Xba*I and *Bam*HI sites, 4.55 kb, Kan^r^	This study
pUTDFZ	∼1.1 kb *Dr-ftsZ* cloned in pUT18 at *Bam*HI and *Kpn*I sites, Amp^r^, 4.12 kb	This study
pUTCDFA	∼1.5 kb *Dr-ftsA* cloned in pUT18C at *Bam*HI and *Kpn*I sites. Amp^r^, 4.55 kb	This study
pUTCEFA	∼1.2 kb *Ec-ftsA* cloned in pUT18C at *Xba*I and *Bam*HI sites. Amp^r^, 4.26 kb	This study
pUTCheA	Chemotaxis protein A cloned in pUT18. Amp^r^	[48]
pKTCheA	Chemotaxis protein A cloned in pKT25. Kan^r^	[48]
pVHSgfp	*gfp* cloned at *ScaI* and *SalI* sites in pVHS559	This study

### Purification of recombinant proteins

**Figure 1:**
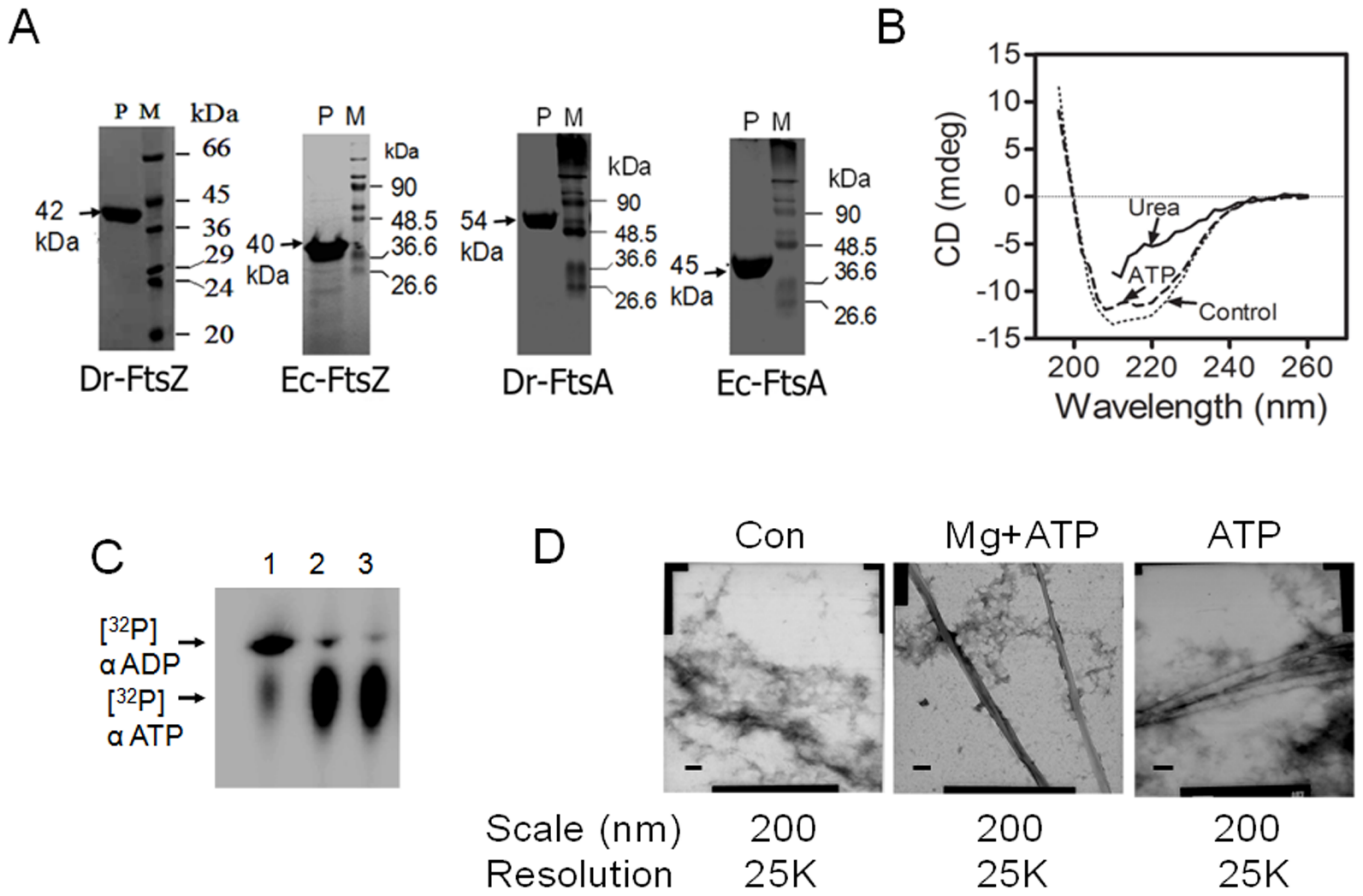
Characterisation of recombinant FtsA of *Deinococcus radiodurans*. Recombinant FtsZ and FtsA of *D. radiodurans* (Dr-FtsZ, Dr-FtsA) and *Escherichia coli* (Ec-FtsZ, Ec-FtsA) were purified to homogeneity (**A**). Dr-FtsA was checked for typical secondary structure characteristics by Circular Dichroism in the absence (Control) and presence of 10 µM ATP (ATP) and in the presence of 8 M Urea (Urea) (**B**). [α32P]-ATP was incubated with recombinant DR1775 as a positive control (1) and in the absence (2) and presence (3) of Dr-FtsA and hydrolysis of [α32P]-ATP substrate ([^32^P]α ATP) into [α32P]-ADP product ([^32^P]α ADP) was monitored on Thin Layer Chromatography (TLC) and autoradiogram was developed as described in methods (**C**). Effect of ATP on polymerization characteristic of Dr-FtsA (Con) was checked in the presence of Mg^2+^ (Mg) and Mg^2+^ + ATP (Mg+ATP) using transmission electron microscopy (**D**). Data shown in (**B**), (**C**) and (**D**) are the representative of the reproducible experiments repeated three times.

Dr-FtsZ was purified from recombinant *E. coli* BL21 (DE3) pLysS as described earlier [20]. Ec-FtsZ was purified from *E. coli* cells expressing recombinant protein using protocols as described in [27]. Recombinant FtsA of both *D. radiodurans* and *E. coli* were purified from *E. coli* TOP10 cells expressing these proteins under *araBAD* promoter on pBADDFtsA and pBADEFtsA plasmids respectively, using metal affinity purification protocols as described in manufacturer's kit (QIAGEN, Germany). In brief, the overnight grown culture of *E. coli* TOP10 harbouring the above constructs was diluted 1∶100 in fresh LB broth and incubated at 37°C at 180 rpm till OD at 600 nm reached 0.3–0.4. The protein was then over expressed by induction with 0.2% arabinose for 3 h. Cells were harvested by centrifugation and lysed in buffer A (50 mM Tris-Cl pH 7.6, 300 mM NaCl, 10% glycerol, 0.5 mM β-mercaptoethanol) containing 2 mM EDTA, 0.5% Triton X100, 0.1% Na-sarcosine, 0.5 mg/ml of lysozyme and 200 µl of phosphatase inhibitor cocktail (Sigma Chemical Company) on ice for 1 h. The cell suspension was sonicated at 25% amplitude with duty cycle of 5 s ‘ON’ and 20 s ‘OFF’ for 5 min. The cell lysate was centrifuged at 11000 rpm for 45 min at 4°C. The clear supernatant containing this protein was dialysed in buffer A containing 10 mM imidazole at 4°C. The dialysed supernatant was passed through pre-charged Ni column pre-equibrated with buffer A containing 10 mM imidazole. The column was then washed extensively with buffer A containing 50 mM imidazole to remove non specifically bounded proteins. Elution was done with 100 mM and 250 mM imidazole buffer in steps and purity was checked on SDS-PAGE. Purification using metal affinity chromatography was repeated. Fractions containing >95% purity were pooled and dialysed in buffer A and the histidine tag was removed as described earlier [20]. The purified protein was concentrated with a10 kDa cut off spin column and then centrifuged at 16000 rpm for 60 min to remove aggregates. Protein was dialyzed in buffer containing 50 mM Tris-Cl pH 7.6, 100 mM KCl, 0.5 mM β-mercaptoethanol, 50% glycerol and 1 mM PMSF and stored at −20°C. Protein concentration was determined by Bradford assay. Thus, recombinant Dr-FtsZ (42 kDa), Ec-FtsZ (40 kDa), Ec-FtsA (45 kDa) and Dr-FtsA (54 kDa) were purified to homogeneity (Fig. 1A) and used in subsequent experiments.

### Immunodetection of protein-protein interaction *in vitro*

Recombinant Dr-FtsZ, Dr-FtsA, Ec-FtsZ and Ec-FtsA proteins purified as above were used for *in vitro* protein-protein interactions studies by following the protocol as described in [28]. In brief, different concentrations of Dr-FtsA, Ec-FtsA and BSA taken as negative control were spotted on nitrocellulose membrane in duplicate and allowed to air dry at room temperature for 2 h. One set was stained with Ponceau red and other set was used for immunoblotting. For later the membrane was wet in buffer B (50 mM Tris-Cl pH 7.6, 150 mM NaCl, 3% glycerol, 0.05% IGEPAL (Sigma Aldrich, USA) and then incubated in blocking solution (buffer B+3% skimmed milk powder) for 1 h. Blots were washed with buffer B and re-soaked overnight in 10 ml of buffer B containing Dr-FtsZ or Ec-FtsZ (1 µg/ml) and incubated with shaking at 4°C. Blots were washed with buffer B and incubated for 2 h at room temperature in blocking solution containing anti-Dr-FtsZ or anti-Ec-FtsZ antibody at 1∶50000 dilutions. The membrane was washed with buffer B and incubated for 2 h at room temperature in blocking solution containing anti-rabbit IgG conjugated with alkaline phosphatase. The membrane was washed extensively with buffer B and developed with NBT-BCIP substrate overnight.

### *In vivo* interaction studies

*In vivo* protein-protein interaction was studied using BACTH two hybrid system (BTH) [29]. For that, *fts*Z and *fts*A from both *E. coli* and *D. radiodurans* were cloned in bacterial two-hybrid plasmids pUT18, pUT18C and pKNT25 and a series of recombinant plasmids were generated as given in Table 2. These constructs were cotransformed in *E. coli* BTH101 host in different combinations. Possible interactions between co-expressing recombinant proteins if any, were detected by monitoring the expression of *lacZ* under cAMP by plate assay and by measuring β galactosidase activity as described earlier [30], [31]. The β galactosidase activity was calculated in Miller Units as described in [32].

### Enzyme activity assay

The GTPase activity of Dr-FtsZ was measured as described earlier [20]. ATPase activity of FtsA was measured using a slightly modified version of the malachite green assay as described in [33]. In brief, 2 µM to 10 µM Dr-FtsA was incubated in buffer PB (25 mM PIPES-HCl pH 7.5, 50 mM KCl) supplemented with 2 mM MgCl_2_ at 37°C for 10 min before the reaction was initiated with 1 mMATP. At required time, the reaction was terminated using 200 µl of freshly prepared malachite green reagent and final volume was made to 1 ml with distilled water. The samples were further incubated at room temperature for 15 min and absorbance at 630 nm was measured against the buffer control and normalized with a protein control that contained all components except ATP as well as ATP control that contained all components except the protein. Amount of Pi released per mole of ATP by unit amount of enzyme was calculated using linear standard curve. To study the effect of FtsA on FtsZ, both the proteins at required concentration were preincubated in buffer PB containing 2 mM MgCl_2_ in presence and absence of 1 mM ATP. Reaction was incubated at 37°C for 10 min. 1 mM GTP was then added and incubation continued for 10 min. Reaction was stopped by addition of freshly prepared malachite green reagent.

ATPase and GTPase activity assay of Dr-FtsA and Dr-FtsZ using radio labeled nucleotides were carried out as described in [34]. In brief, the recombinant proteins, 10 µM each were preincubated in buffer PB in the presence of 2 mM MgCl_2_ and 0.5 mM ATP/0.5 mM GTP for monitoring GTPase and ATPase activity respectively for 10 min at 37°C. To these, 0.5 mM GTP and 30 nM [α32P]-GTP or 0.5 mM ATP and 50 nM [α32P]-ATP were added and reaction mixtures were further incubated at 37°C for 10 min. Recombinant DR1775 was found to have ATPase activity was used as positive control (A. D. Das and H. S. Misra, unpublished data). 2 µl of the reaction mixture was spotted on PEI-Cellulose F^+^ TLC sheets. Spots were dried and mixtures were separated on solid support in buffer system in 0.75 M KH_2_PO_4_/H_3_PO_4_ pH 3.5. Autoradiogram images were developed and documented on phosphorimager (Molecular Dynamics, Inc).

### Sedimentation analysis

The sedimentation assay was carried out using modified protocol as described earlier [20]. In brief, the recombinant proteins were incubated in buffer PB in the presence of 2 mM MgCl_2_ for 10 min at 37°C and centrifuged at 22000×g for 10 min. Supernatant containing soluble protein was estimated for concentration and used for further studies. 5 µM purified recombinant FtsZ was incubated with different molar ratios of Dr-FtsA in PB buffer with 1 mM ATP and/or GTP in different combination. Mixture was preincubated at 37°C for 10 min, 1 mM GTP was added and immediately centrifuged at 150 000×g for 15 min at 37°C, using Optima MAX XP ultracentrifuge (Beckman, Inc). The pellets and supernatants were separated and analyzed by SDS-PAGE. Protein bands were visualized by coomassie blue staining. Band intensity was quantified by Gelquantnet software.

### Fluorescence microscopic studies

Fluorescence microscopic studies were carried out essentially as described in [20], [23]. In brief, overnight grown culture of *E. coli* MG1655 containing pBADHisA + pVHSgfp, pBADHisA + pFTSZGFP, pBADDftsA + pVHSgfp and pFTSZGFP + pBADDftsA were diluted 1∶100 in fresh LB containing 100 µg/ml ampicillin and 50 µg/ml spectinomycin and incubated at 37°C at 180 rpm till OD reached 0.3–0.4. Induction was done with either 0.2% arabinose or 0.5 mM IPTG or both as required for 2 h. Expression of recombinant fusion protein was confirmed by immunoblotting using antibodies against either Dr-FtsZ or polyhistidine tag using a protocol as described earlier [20]. Cells expressing recombinant proteins were collected and washed once with phosphate buffered saline (PBS). The cell pellet was then suspended in PBS, and immobilized on agar pad made on a slide and observed using a fluorescence microscope (Model AXIOIMAGER M1, Carl Zeiss, Germany). Cell length was measured using Axiovision 4.8 software. Image processing was done using Adobe Photoshop version 4.0 and Image J software and data was analysed for statistical parameters using Graphpad prism software.

### Electron Microscopy and Circular Dichroism spectral studies

Transmission electron microscopy (TEM) was carried out for monitoring the polymerization characteristic of purified recombinant Dr-FtsA using electron microscope (Model JEOL2000FX, Japan) as described previously [20]. In brief, 5 µM of Dr-FtsA was preincubated in buffer 25 mM Pipes pH 7.5, 50 mM KCl with and without 5 mM MgCl_2_ for 5 min at 37°C. Then 1 mM ATP was added as required and further incubated at 37°C for 5 min. 10 µl of sample was spotted on 200 mesh size copper grid and excess blotted dry. After 5 min, staining was carried out with 1% uranyl acetate and excess solution blotted dry. The grid was kept at RT for 1 h before imaging using an electron microscope.

Circular Dichroism spectra were recorded for Dr-FtsA dialysed in phosphate buffer using JASCO, J815, Japan as described earlier [20]. In brief, 5 µM of Dr-FtsA was incubated in phosphate buffer with and without ATP and then subjected to CD analysis. Protein incubated with 8 M Urea was used for recording CD spectra. CD was recorded at 37°C. Ellipticities were corrected for buffer contribution and the final data plotted.

All experiments were repeated at least three times and results were reproducible. Data presented without standard deviations are illustrative of a typical experiment and represent the average of three replicates wherein the variation among replicates was less than 10%.

## Results

### Dr-FtsA lacks ATPase activity but requires ATP for polymerization

FtsA is a member of actin/HSP70/sugar kinase superfamily of ATP binding proteins in bacteria. The effect of ATP on FtsA interaction with FtsZ has also been shown in gamma proteobacteria [2]. For functional characterization, recombinant Dr-FtsA was purified from *E. coli* (Fig. 1A). Circular Dichroism (CD) spectral analysis of purified protein indicated that Dr-FtsA used in this study had the correct secondary structure assignment, and interacted with ATP *in solution* (Fig. 1B). The purified protein was checked for ATPase activity as well as polymerization in the presence of ATP *in vitro*. Dr-FtsA did not show ATP hydrolysis even up to 10 µM protein while a positive control could convert ATP into ADP (Fig. 1C). Polymers of recombinant Dr-FtsA were examined using a transmission electron microscope (TEM). It was observed that the protein formed amorphous structures presumably, due to random interaction of molecules in the presence of Mg^2+^. However, the presence of Mg^2+^ and ATP together, as well as ATP alone stimulated Dr-FtsA polymerization to a filamentous structure (Fig. 1D). FtsAs from some other bacteria also lack ATPase activity but were able to polymerize [10], [11]. This indicated that Dr-FtsA is functional but does not have ATPase activity, and its ordered polymerization requires Mg^2+^ and ATP. Therefore, the effect of Dr-FtsA on Dr-FtsZ functions was evaluated in the presence and absence of ATP.

### Dr-FtsA differentially affects Dr-FtsZ and Ec-FtsZ functions *in vitro*

**Figure 2:**
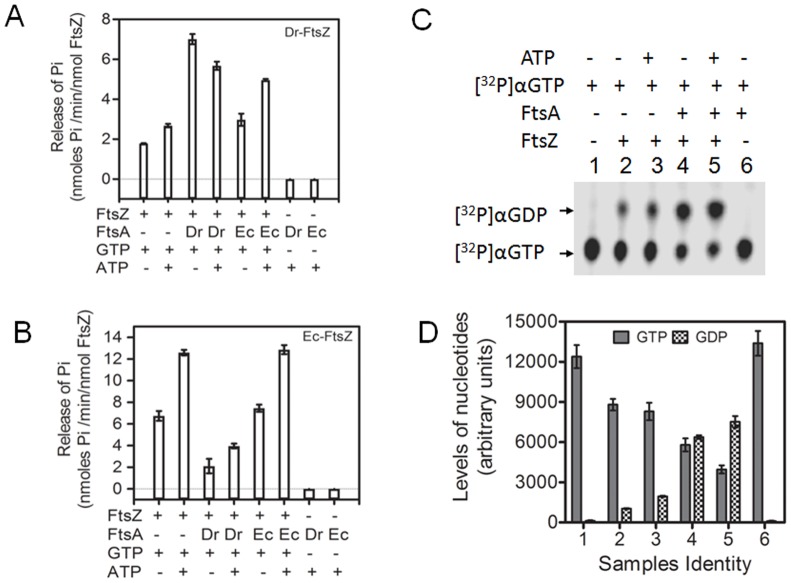
FtsA effect on GTPase activity of FtsZ from *Deinococcus radiodurans* as well as *Escherichia coli*. Purified recombinant Dr-FtsZ and Ec-FtsZ were incubated in different combinations with GTP, ATP, Dr-FtsA (Dr) and Ec-FtsA (Ec) and the release of inorganic phosphate (Pi) was monitored (**A, B**). Similarly, Dr-FtsA (FtsA), Dr-FtsZ (FtsZ), ATP and [α 32P] GTP were incubated in different combinations and conversion of [α32P]-GTP substrate ([^32^P]α GTP) into [α32P] GDP product ([^32^P]α GDP) was monitored on TLC and autoradiogram was developed (**C**). Levels of both substrate (GTP) and product (GDP) were determined densitometrically for samples numbered as 1–6 as shown in panel C and plotted (**D**).

The effect of Dr-FtsA on the GTPase activity and polymerization of recombinant Dr-FtsZ and Ec-FtsZ was monitored. Dr-FtsA showed a differential effect on FtsZ from *D. radiodurans* and *E. coli*. For instance, GTPase activity of Dr-FtsZ was stimulated in the presence of Dr-FtsA (Fig. 2A and Fig. 2C). Purified recombinant Ec-FtsA also stimulated the GTPase activity of Dr-FtsZ but to a lesser extent than Dr-FtsA (Fig. 2A). In the presence of ATP, the stimulation of GTPase activity of Dr-FtsZ by Ec-FtsA or Dr-FtsA was found to be different. While ATP increases the Ec-FtsA stimulated DrFtsZ GTPase activity, Dr-FtsA stimulation of Dr-FtsZ GTPase activity was marginally reduced in the presence of ATP (Fig. 2A). In the case of Ec-FtsZ, the GTPase activity was decreased by nearly 3 fold in the presence of Dr-FtsA (Fig. 2B), which was not affected by the presence of Ec-FtsA. Further, ATP did not influence the effect of either FtsAs on the GTPase activity of Ec-FtsZ. However, the GTPase activity of Dr-FtsZ was stimulated in the presence of ATP (Fig. 2C), which was found to be in agreement with the results reported recently [20]. This indicated that Dr-FtsA functions differently with FtsZ of *D. radiodurans* and *E. coli* and ATP effect on FtsA function from these bacteria is also different.

**Figure 3:**
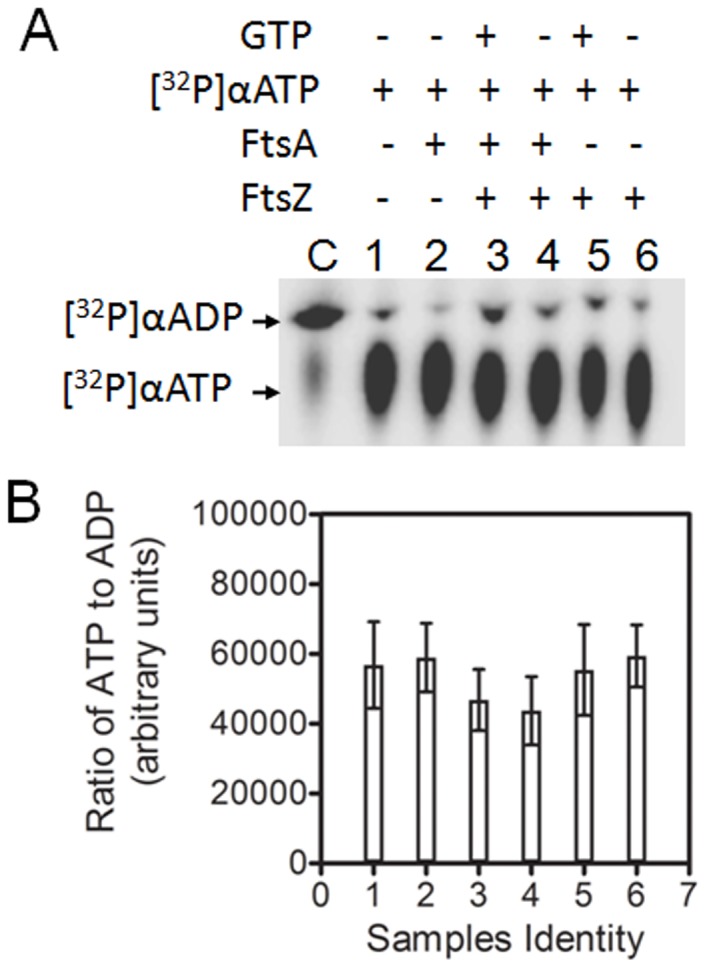
ATP hydrolysis by Dr-FtsA and Dr-FtsZ in different combinations. Purified recombinant Dr-FtsA and Dr-FtsZ were incubated with GTP and [α32P]-ATP substrate in different combinations. [α32P]-ATP ([^32^P]α ATP) hydrolysis to [α32P]-ADP ([^32^P]α ADP) in different samples (1–6) was monitored on TLC and autoradiogram was developed. Conversion of [α32P]-ATP into [α32P]-ADP was compared with recombinant DR1775 having ATPase activity as positive control (C) as described in methods (**A**). Band intensities of samples (1–6) corresponding to panel A were determined densitometrically, ATP/ADP ratios were calculated and plotted as histograms (**B**).

Earlier it was shown that Dr-FtsZ does not hydrolyse ATP and that ATP stimulates GTP hydrolysis by Dr-FtsZ. Here, we showed that Dr-FtsA has neither ATPase nor GTPase activity and that the release of inorganic phosphorous (Pi) increased when Dr-FtsZ was incubated with GTP and ATP together, or with Dr-FtsA. Therefore, the possibility that Dr-FtsA or Dr-FtsZ able to hydrolyse ATP upon their interaction had to be ruled out. Hence, the ATPase activity was measured using [α32P]-ATP as a substrate in different combinations of these enzymes with nucleotides. Results showed that neither Dr-FtsZ nor Dr-FtsA alone or in combination could hydrolyse ATP (Fig. 3). Since, Dr-FtsA could not hydrolyse GTP (Fig. 2C); its stimulation of GTPase activity of Dr-FtsZ was strongly supported. These results provided evidence that Dr-FtsA has neither ATPase nor GTPase activity in both isolated form as well as when it is present together with Dr-FtsZ. Therefore, the stimulation of Dr-FtsZ GTPase in the presence of either ATP or Dr-FtsA would be due to direct influence of these factors on Dr-FtsZ functions.

**Figure 4:**
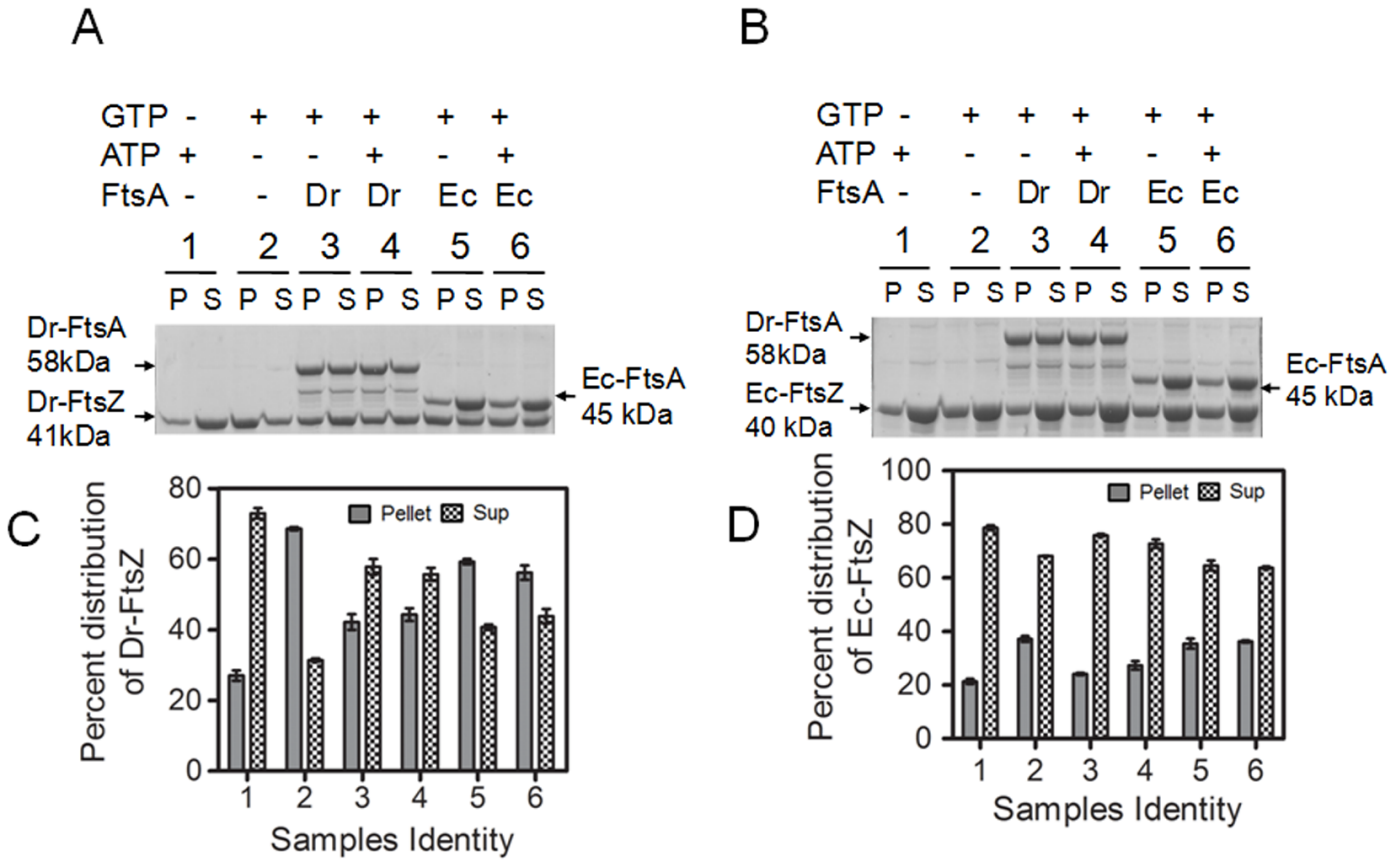
FtsA effects on sedimentation characteristics of FtsZ from *Deinococcus radiodurans* and *Escherichia coli*. Purified recombinant Dr-FtsZ (**A**) and Ec-FtsZ (**B**,) were incubated in different combinations with GTP, ATP, Dr-FtsA (Dr) and Ec-FtsA (Ec) and sedimentation characteristics of FtsZs were monitored as described in methods. Both pellets and supernatant fractions were analysed on SDS-PAGE and coomassie stained protein bands were quantified densitometrically and depicted in bar diagrams (**C, D**). Sample identity (1–6) in panel C and D is similar to panel A and B, respectively.

Since the GTPase activity of FtsZ influences its polymerization-depolymerization dynamics, the effect of FtsA on polymerization was monitored by sedimentation. We observed that the levels of Dr-FtsZ in the pellet decreased significantly in the presence of both Dr-FtsA and Ec-FtsA, independent of the presence of ATP (Fig. 4A). On the other hand, the amount of Ec-FtsZ in the pellet remained nearly unchanged in the presence of Ec-FtsA while it decreased significantly in the presence of Dr-FtsA as compared to its absence (Fig. 4B). This might indicate that Dr-FtsA possibly interferes with Ec-FtsZ polymerization.

**Figure 5:**
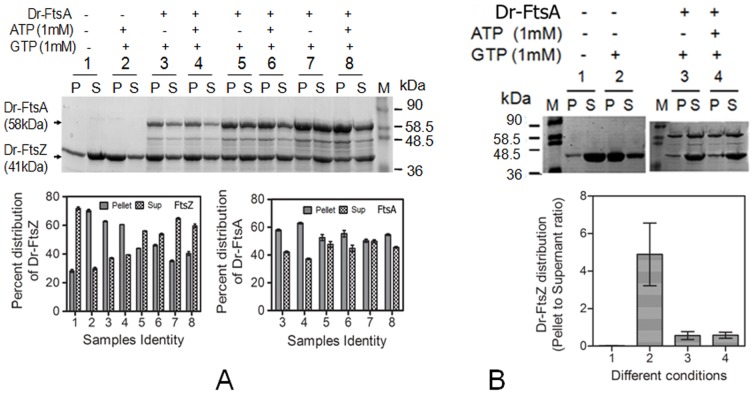
Concentration effect of Dr-FtsA on sedimentation characteristic of Dr-FtsZ. **A**. Purified recombinant Dr-FtsZ was incubated with increasing molar ratio of Dr-FtsA such as 1∶0.5 (3,4), 1∶1 (5,6) and 1∶2 (7,8) under standard assay conditions of Dr-FtsZ described elsewhere in this paper. Samples were spun at 150 000×g for 15 min at 37°C. Both pellet and supernatant fractions obtained were analysed on SDS-PAGE (upper panel) and protein bands corresponding to Dr-FtsZ (FtsZ) and Dr-FtsA (FtsA) were quantified densitometrically for samples numbered as 1-6 and presented as histogram (lower panel) **B**. Dr-FtsZ (1) was incubated with both 2 mM Mg^2+^ and 1 mM GTP (2, 3, 4) for 10 min and then purified Dr-FtsA was added in 1∶1 molar ratio and further incubated for another 10 min in the absence (3) and presence (4) of ATP. Samples (1–4) were spun at 22000×g for 1 h and corresponding pellet and supernatants were analysed on SDS-PAGE (upper panel). Density of FtsZ protein bands in these samples were plotted as histograms (lower panel).

The productive interaction of FtsA and FtsZ is regulated by stoichiometric ratio of these proteins in the cell which differs from bacterium to bacterium [9], [12]. Therefore, the molar ratio of Dr-FtsZ to Dr-FtsA that favours reduction of Dr-FtsZ in pellet presumably through depolymerization was determined. For that, the sedimentation of Dr-FtsZ was monitored by co-incubating it with Dr-FtsA in different molar ratios. Significant increase in the amount of Dr-FtsZ in supernatant was observed when Dr-FtsA and Dr-FtsZ were present in 1∶1 molar ratio as compared with the control (Fig. 5A). These results suggested that the presence of Dr-FtsA in a correct molar ratio can influence FtsZ polymerization dynamics and thus GTPase activity. In order to rule out the inhibitory effect of Dr-FtsA on Dr-FtsZ polymerization and to confirm that indeed Dr-FtsA was stimulating GTPase activity which was leading to depolymerization, Dr-FtsZ was first allowed to polymerize and then Dr-FtsA was added in the presence and absence of ATP. Protein distribution between pellet and supernatant was monitored through sedimentation analysis. An increase in Dr-FtsZ amount was observed in the supernatant even when Dr-FtsA was added after Dr-FtsZ had polymerised (Fig. 5B). This indicated that Dr-FtsA stimulates GTPase activity of Dr-FtsZ and that possibly induces its depolymerisation *in vitro.* Thus, these results suggested that Dr-FtsA regulates GTPase activity and polymerization dynamics of Dr-FtsZ and Ec-FtsZ differently and is seems to be independent of ATP hydrolysis.

### Dr-FtsA differentially interacts with Dr-FtsZ and Ec-FtsZ

**Figure 6:**
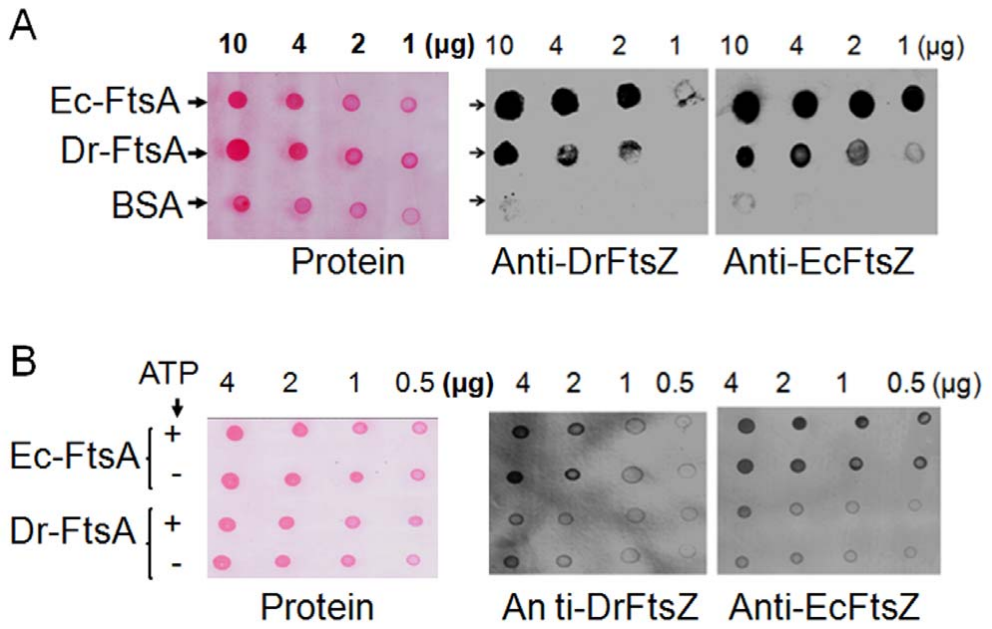
*In vitro* interaction of FtsZ with FtsA. Increasing concentration (1–10 µg) of Dr-FtsA and Ec-FtsA were incubated in the absence (**A**) and presence of ATP (**B**) for 10 min and blotted on nitrocellulose support (Protein). Blots were incubated separately with 1 µg/ml of purified recombinant Dr-FtsZ and Ec-FtsZ overnight at 4°C as described in methods, washed gently and hybridized with antibodies against Dr-FtsZ (Anti-DrFtsZ) and Ec-FtsZ (Anti-EcFtsZ), respectively. Signals were detected using NBT/BCIP system for alkaline phosphatase. BSA was taken as a control and processed in parallel. Data shown are representatives of the reproducible experiments repeated three times.

The *in vitro* interactions between purified Dr-FtsZ, Dr-FtsA, Ec-FtsZ and Ec-FtsA proteins were monitored by immobilizing FtsA on a membrane and probing this membrane with FtsZ *in solution* as detailed in methods. FtsZ bound to FtsA was detected by immunoblotting using antibodies against Dr-FtsZ and Ec-FtsZ. We observed that FtsZ from both *D. radiodurans* and *E. coli* interacted with Dr-FtsA and Ec-FtsA (Fig. 6A). Further, ATP did not produce significant change in FtsZ interaction with FtsA particularly when immobilized on solid support (Fig. 6B). These results might suggest that Dr-FtsA could interact with Dr-FtsZ and Ec-FtsZ *albeit* to different levels, at least *in vitro*. Therefore, *in vivo* interactions of these proteins were studied using bacterial two-hybrid system [35]. The expression of β-galactosidase, which, could have occurred only when two proteins would have interacted in the cells, was monitored.

**Figure 7:**
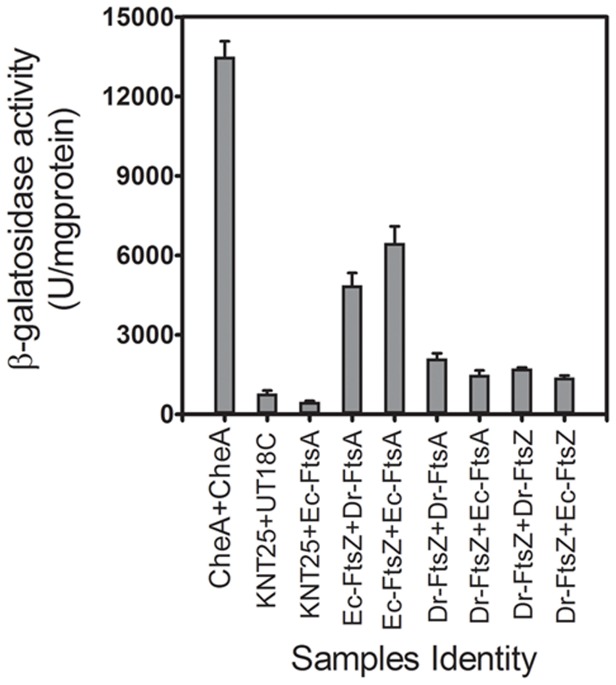
*In vivo* interaction of FtsA with FtsZ using bacterial two hybrid system. *Dr-ftsA*, *Ec-ftsA*, *Dr-ftsZ* and *Ec-ftsZ* were cloned in bacterial two hybrid system plasmids as described in methods and given in Table 2. These were cotransformed into *E. coli* BTH101 in different combinations. Expression of β-galactosidase was assayed in liquid culture of these cells grown overnight in presence of IPTG and activity calculated as described in methods. Cells co-transformed with both the vectors (KNT25+UT18C), with one vector and one construct (KNT25+Ec-FtsA) were used as negative control while CheA subunit expressing on both the vectors (CheA+CheA) was used as positive control.

We observed that the cells co-expressing Ec-FtsZ with either Dr-FtsA or Ec-FtsA produced intense blue colour colonies on LB plate containing 5-bromo-4-chloro-3-indolyl β-D-galactopyranoside (X-gal) indicating the expression of β-galactosidase in these cells (Data not shown). Cells co-expressing Dr-FtsZ and FtsA from either *E. coli* or *D. radiodurans* also showed β-galactosidase expression, but the level of expression was much less as compared to the cells co-expressing Ec-FtsZ with any of these FtsAs. Cells co-harbouring respective vectors produced colourless colonies while positive control expressing CheA subunits produced intense blue colour colonies. Levels of β-galactosidase activity in these cells were in agreement with the blue colour intensity in spot assay (Fig. 7). These results showed the *in vivo* interaction of Ec-FtsZ with Dr-FtsZ. The interaction of Ec-FtsZ and Ec-FtsA has already been reported earlier [36]. These results suggested that both Ec-FtsZ and Dr-FtsZ could interact with FtsA from both *E. coli* and *D. radiodurans.* However, Ec-FtsZ interaction with either FtsAs is relatively stronger than Dr-FtsZ, at least in *E. coli* host.

### The effect of Dr-FtsA on cell division in *E. coli* was not restored upon co-expression of Dr-FtsZ

**Figure 8:**
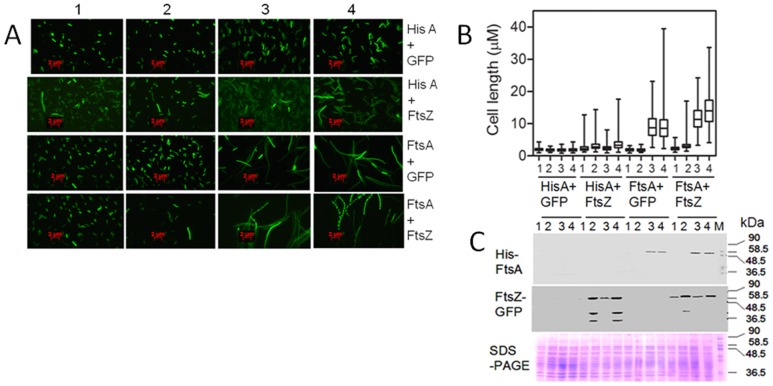
Effect of Dr-FtsA and Dr-FtsZ over expression on cell division in *Escherichia coli*. *E. coli* MG1655 was transformed with vectors pBADHisA and pVHSGFP (HisA+GFP), pBADHisA and pFTSZGFP (HisA+FtsZ), pBADDftsA and pVHSgfp (FtsA+Gfp), and pBADftsA and pFTSZGFP (FtsA+FtsZ). These cells were grown in the absence (1) and presence of IPTG (2), arabinose (3) and both IPTG and arabinose (4) for 3 h. These cells were micrographed (A) and cell lengths of nearly 500 independent cells were measured and presented as histograms (**B**). Aliquots were taken and nearly equal amount of total proteins were analysed on SDS-PAGE (SDS-PAGE) and blotted with antibodies against (His)6 tag (His-FtsA) and Dr-FtsZ(GFP-FtsZ) (**C**).

Dr-FtsA was expressed in *E. coli* from the pBADDftsA plasmid under arabinose inducible promoter. Similarly, Dr-FtsZ-GFP fusion was expressed in *E. coli* on pFTSZGFP under IPTG inducible promoter. These cells were imaged microscopically and cell length was measured. Unlike controls, *E. coli* cells expressing either Dr-FtsA or Dr-FtsZ-GFP showed cell elongation (Fig. 8A) and formed tiny colonies (data not shown). The cell length in Dr-FtsA expressing cells was greatly increased compared to cells expressing Dr-FtsZ (Fig. 8B). These results indicated that *in trans* expression of either Dr-FtsZ or Dr-FtsA could affect cell division in *E. coli*. Since, FtsA is the first divisome component to be recruited to the Z-ring and acts in tandem with FtsZ during Z ring formation, titration of Ec-FtsZ from ring formation by either Dr-FtsA or Dr-FtsZ could explain this observation. Hence, the effect of Dr-FtsZ over expression on cell division inhibition in Dr-FtsA overexpressing *E. coli* cells was checked. Dr-FtsZ-GFP and Dr-FtsA were co-expressed in *E. coli* and their expression was confirmed (Fig. 8C). We observed that co-expression of Dr-FtsA and Dr-FtsZ-GFP did not rescue *E. coli* from cell elongation caused due to overexpression of both these proteins individually (Fig. 8). These results suggested that cell elongation caused due to overexpression of FtsA and FtsZ from *D. radiodurans* was possibly due to inhibition of *E. coli* cell division proteins.

## Discussion

Bacterial cell division is regulated by coordinated action of several proteins involved in cytokinesis and genome maintenance [37]. FtsZ ring formation and its dynamics that eventually lead to cytokinesis is both spatially and temporally regulated. FtsZ localization is regulated by ‘Min’ proteins and the proteins that are involved in nucleoid occlusion mechanism [38]. It is temporally regulated by the processes linked to DNA repair and genome segregation [37]. The FtsZ ring dynamics at the division site is under the influence of various proteins that co-assemble to form the divisome [39]. These proteins individually modulate the polymerization of FtsZ by either stabilizing or destabilizing effect. The net effect is the formation of a functional Z-ring. FtsA is one of the initial proteins to be recruited to the Z-ring [39]. It not only helps in tethering the Z-ring to the membrane but also control its dynamics [1]. This has been very well demonstrated in case of Ec- FtsA and Ec-FtsZ using total internal reflection fluorescence microscopy [2].

**Figure 9:**
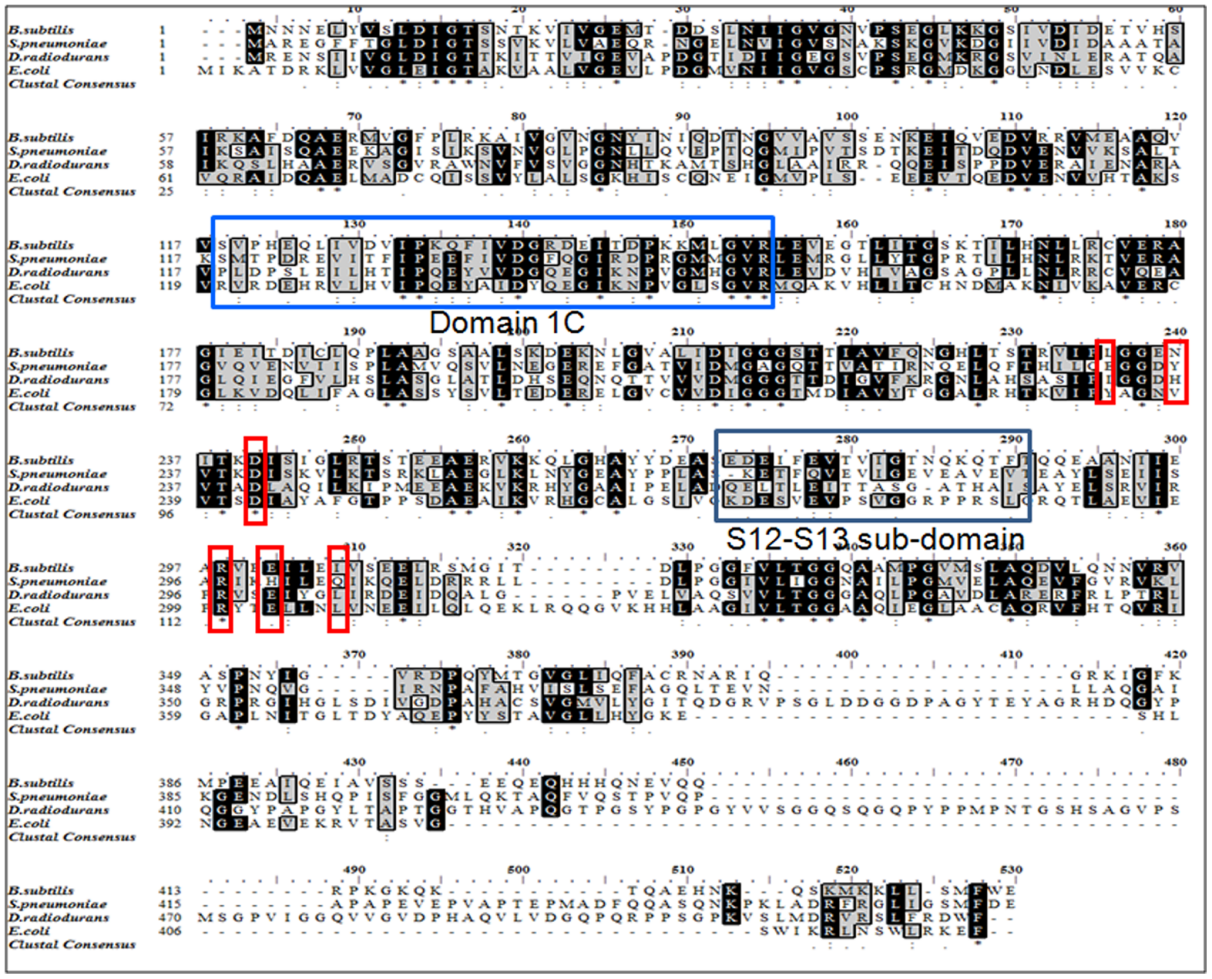
Multiple sequence alignment of FtsA homologues with Dr-FtsA. Amino acid sequence of FtsA (DR_0630) of *D. radiodurans* (D. radiodurans) was aligned with its homologs from *Bacillus subtilis* (B. subtilis), *Streptococcus pneumoniae* (S.pneumoniae) and *Escherichia coli* (E. coli). Conserved residues in the domain IC and S12–S13 motifs are boxed with blue colour and other residues that are found to be important for FtsA interaction with FtsZ are boxed with red colour.

Dr-FtsZ shows 40–60% amino acid identity while amino acid identity for Dr-FtsA was between 30–45% with their respective homologues. The functional domains of these proteins are highly conserved along with the marked differences in their terminal regions (Fig. 9). For instance, Dr-FtsA has a nearly conserved 1C subdomain and S12–S13 motifs of sub-domain 2B when compared with its homologues including Ec-FtsA. The S12–S13 motifs in sub-domain 2B are involved in FtsA-FtsZ interaction in *E. coli* as well as in *T. maritima* [8], [10] and sub-domain 1C is involved in homodimerization of FtsA. This could explain Dr-FtsA interaction with Ec-FtsZ and Dr-FtsZ, and the polymerizing ability of Dr-FtsA as observed in sedimentation (Fig. 5A) and TEM (Fig. 1D) studies. Also Dr-FtsA stimulation of GTPase activity of Dr-FtsZ thus affecting its polymer stability points out to the role of FtsA in regulating the dynamics of FtsZ. Recently, FtsA from *S. aureus* was also shown to stimulate the GTPase activity of its FtsZ [40].

The *Deinococcus radiodurans* genome does not reveal the presence of many accessory divisome components that play important roles in FtsZ polymerization dynamics in other bacteria [18]. Hence the role of FtsA in this bacterium is likely to be very important. Dr-FtsA has two regions at its C-terminus (amino acids 396–413 and 452–513), which are absent in FtsA of *E. coli*, *B. subtilis* and *S. pneumoniae* (Fig. 9). The possibility of these structural differences contributing to inhibition of GTPase activity of Ec-FtsZ by Dr-FtsA cannot be ruled out. Interactions between *E. coli* divisome components have been demonstrated using the bacterial two hybrid system [35]. Using a similar approach as well as *in vitro* studies, we found that Dr-FtsA interacts less efficiently with Dr-FtsZ than Ec-FtsZ, while the Ec-FtsA interaction with Dr-FtsZ was relatively efficient. The possibility of the strong interaction between Dr-FtsA and Ec-FtsZ (Figs. 6 and 7) and Dr-FtsA inhibition of GTPase activity of Ec-FtsZ (Fig. 2C) contributing to cell division inhibition in *E. coli* cannot be ruled out. Factors responsible for Dr-FtsA interaction with Dr-FtsZ in *E. coli* are not understood. One possible explanation could be that these deinococcal proteins expressed in *E. coli* could be qualitatively different than they might actually be when present in *D. radiodurans.* Amongst different factors that could affect the quality of these proteins, post translational modification particularly serine/threonine phosphorylation of proteins can be a contributory factor. Unlike many bacteria including *E. coli*, the *D. radiodurans* genome encodes a large number of Serine/Threonine/Tyrosine protein kinases [41] and the role of one such kinase, RqkA, in the radiation resistance of this bacterium has been demonstrated [42]. Phosphorylation of FtsZ by Pkn kinases in *Mycobacterium tuberculosis* [43] and by an indigenous kinase in *Corynebacterium glutamicum* [44], with an effect on FtsZ characteristics, has been demonstrated. We have observed that some of the cell division proteins including FtsZ and FtsA of *D. radiodurans* are phosphosubstrates for the RqkA kinase *in vitro* (Kruti M Modi and H. S. Misra, unpublished data) and detailed studies on the functional significance of phosphorylation of these proteins in radiation stressed growth regulation of *D. radiodurans* will be reported independently. Recently, we have demonstrated that phosphorylation of PprA, a DNA repair protein, by RqkA affected DNA metabolic functions of this protein and eventually its role in radioresistance [45].

In summary, our findings suggest that Dr-FtsA is functional although it lacks ATPase activity *in vitro* and interacts differentially with Dr-FtsZ and Ec-FtsZ. As a result, it differently regulates the GTPase activity and dynamics of polymerization/depolymerisation of these FtsZ enzymes. The exact mechanism(s) underlying the differential effects of Dr-FtsA on Dr-FtsZ and Ec-FtsZ functions and relatively weak interaction with Dr-FtsZ are yet to be understood. However, the possibilities of Dr-FtsA's sequence similarities, particularly in domain 1C and S12–S13 motifs of sub-domain 2B, with Ec-FtsA, if helping former to interact better with Ec-FtsZ than Dr-FtsZ, and the *in vivo* conditions that are required for efficient interaction of Dr-FtsZ with Dr-FtsA in *D. radiodurans* if different from *E. coli* could be hypothesized and would be worth investigating independently.
